# BIRC5 facilitates cisplatin‐chemoresistance in a m^6^A‐dependent manner in ovarian cancer

**DOI:** 10.1002/cam4.6811

**Published:** 2023-12-19

**Authors:** Yadan Fan, Yinglian Pan, Liping Jia, Shuzhen Gu, Binxin Liu, Ziman Mei, Chunyan Lv, Haohao Huang, Genhai Zhu, Qingchun Deng

**Affiliations:** ^1^ Department of Gynecology The Second Affiliated Hospital of Hainan Medical University Haikou China; ^2^ Department of Oncology The First Affiliated Hospital of Hainan Medical College Haikou China; ^3^ Department of Neurosurgery General Hospital of Central Theater Command of Chinese People's Liberation Army Wuhan China; ^4^ Department of Gynecology Hainan General Hospital, Hainan Affiliated Hospital of Hainan Medical University Haikou China

**Keywords:** BIRC5, chemoresistance, IGF2BP1, METTL3, ovarian cancer

## Abstract

Cisplatin‐based chemotherapy is the standard treatment for metastatic ovarian cancer (OC). However, chemoresistance continues to pose significant clinical challenges. Recent research has highlighted the baculoviral inhibitor of the apoptosis protein repeat‐containing 5 (BIRC5) as a member of the inhibitor of the apoptosis protein (IAP) family. Notably, BIRC5, which has robust anti‐apoptotic capabilities, is overexpressed in numerous cancers. Its dysfunction has been linked to challenges in cancer treatment. Yet, the role of BIRC5 in the chemoresistance of OC remains elusive. In our present study, we observed an upregulation of BIRC5 in cisplatin‐resistant cell lines. This upregulation was associated with enhanced chemoresistance, which was diminished when the expression of BIRC5 was silenced. Intriguingly, BIRC5 exhibited a high number of N6‐methyladenosine (m^6^A) binding sites. The modification of m^6^A was found to enhance the expression of BIRC5 by recognizing and binding to the 3′‐UTR of mRNA. Additionally, the insulin‐like growth factor 2 mRNA‐binding protein 1 (IGF2BP1) was shown to stabilize BIRC5 mRNA, synergizing with METTL3 and intensifying chemoresistance. Supporting these in vitro findings, our in vivo experiments revealed that tumors were significantly smaller in size and volume when BIRC5 was silenced. This reduction was notably counteracted by co‐silencing BIRC5 and overexpressing IGF2BP1. Our results underscored the pivotal role of BIRC5 in chemoresistance. The regulation of its expression and the stability of its mRNA were influenced by m^6^A modifications involving both METTL3 and IGF2BP1. These insights presented BIRC5 as a promising potential therapeutic target for addressing cisplatin resistance in OC.

## Abstract 2

This article focused on the mechanism of chemoresistance in ovarian cancer. First, we found BIRC5 upregulated significantly in cisplatin resistant cells and contributed to chemoresistance. Then, we found that METTL3 and IGF2BP1 coordinated to regulate BIRC5 expression level and mRNA stability in an m^6^A dependent manner. This result confirmed in vivo and vitro.

## 1 INTRODUCTION

Ovarian cancer (OC) is the leading gynecological malignancy worldwide and poses a significant threat to women's health, accounting for the highest mortality rates among gynecological cancers.^1^ In China, OC ranks as the third most common cancer affecting the female reproductive system. In its early stages, OC often remains asymptomatic. By the time symptoms such as abdominal pain, bloating, and irregular vaginal bleeding manifest, the disease is frequently in an advanced stage, correlating with a poor prognosis.^2^ The primary therapeutic approach for OC combines cytoreductive surgery with platinum‐based chemotherapy.^3^ However, despite this treatment regimen, recurrence rates soar as high as 70% within 3 years.^4^ While many patients initially respond well to chemotherapy, others face recurrence after standard treatment. Moreover, with each successive chemotherapy treatment and increased dosage, the intervals between treatments shorten, leading to heightened chemoresistance and a deteriorating prognosis.^5^ Research suggests that heightened antioxidant capacities within cancer cells and increased capabilities to expel drugs are primary contributors to chemoresistance.^6^, ^7^, ^8^, ^9^ Additionally, an enhanced ability to repair DNA damage and the inactivation of tumor cell apoptosis are other prevalent factors.^10^, ^11^ Epigenetic changes also play a pivotal role, often leading to aberrant gene expression.^12^, ^13^ Despite these insights, the specific mechanisms underlying chemoresistance in OC remain elusive.

N6‐methyladenosine (m^6^A) methylation is the most prevalent modification in mRNA and is dynamically reversible, contingent upon specific enzymes. The implications of m^6^A span a wide range, including the regulation of gene expression, modulation of mRNA stability or degradation, splicing, and interactions between RNA and protein. The m^6^A modification is introduced by enzymes known as methyltransferases, referred to as “writers,” and removed by demethylases, termed “erasers.” These are RNA‐binding proteins that either directly or indirectly recognize the m^6^A motif and play roles in RNA stabilization, degradation, and translation.^14^, ^15^ While prior studies have identified the influence of m^6^A in OC progression, its role in OC chemoresistance remains elusive. Baculoviral IAP repeat containing 5 (BIRC5) has been identified as a member of the inhibitor of the apoptosis proteins (IAP) family.^16^ Although it has a low expression level in healthy adult tissues, BIRC5 is markedly upregulated in a variety of tumors, where it assumes an oncogenic role. Its expression is modulated by circadian rhythms and is activated by transcription factors such as STAT, NF‐κB, and other signal transducers.^17^ Several studies have reported that miRNAs, small‐molecule inhibitors, antisense oligonucleotides, and peptide‐based immunotherapy can significantly downregulate BIRC5 expression, consequently inhibiting tumor cell growth.^18^ Nonetheless, the biological significance of BIRC5 and its regulatory mechanisms related to drug resistance in OC remain unclear.

In this study, we conducted a series of in vitro and in vivo functional experiments to investigate the role and regulation of the BIRC5 gene in OC with cisplatin resistance. Our findings illuminated the biological significance of m^6^A modifications mediated by METTL3 and IGF2BP1. These entities controlled the expression of BIRC5 and its mRNA stability, respectively. Consequently, our results suggested that BIRC5 might serve as a potential therapeutic target for addressing chemoresistance in OC.

## 2 METHODS

### 2.1 Establishment of stable cell lines

Ovarian carcinoma cell lines SKOV3 and A2780 were obtained from American Type Culture Collection (ATCC). Cells cocultured with gradually increase the concentration of cisplatin, and then obtained SKOV3/DDP and A2780/DDP after 6 months. To identify sensitive and resistant cells using CCK8 and the median inhibitory concentrations (IC_50_). SKOV3 and SKOV3/DDP cultured in McCoy's 5A medium, while A2780 and A2780/DDP cultured in DMEM medium, supplemented with 10% FBS (Gibco, ThermoFisher, USA), 100 μg penicillin and 100 U/mL streptomycin (Pricella, China). All cells were cultured at 37°C in a humidified atmosphere of 5% CO_2_.

### 2.2 Cell viability and IC_50_

Cell viability detected by CCK8 assay. SKOV3, SKOV3/DDP, A2780, A2780/DDP were seeded 2000 cells into 96‐well templates for 24 h, and treated with different doses (0, 5, 10, 20, 40, and 80 μM) of cisplatin for another 24 h, then added 10 μL CCK8 to incubate for 3 h. Absorbance was examined at 450 nm using a microplate reader (BioTek, USA).

### 2.3 Western blotting

OC cells were washed twice in pre‐chilled PBS, and lytic cells in RIPA lysis buffer with protease and phosphatase inhibitors. Protein lysate were centrifuged at 10,000 *g*, and supernatants were denatured in 100°C with 1 × loading buffer (Biosharp, China), and the protein concentration was determined using BCA method. Protein samples were separated by sodium dodecyl sulfate‐polyacrylamide gel electrophoresis (SDS‐PAGE) and transferred to PVDF membranes. These PVDF membranes were closed in 5% nonfat dried milk (Biosharp, China), and hatch to primary antibodies overnight at 4°C, which including: BIRC5 (1:1000, Abcm, ab134170), METTL3 (1:1000, Abcam, ab195352), IGF2BP1 (1:1000, Abcam, ab184305), GAPDH (1:1000, Abcam, ab128915), METTL14 (1:1000, Abcam, 300,104), METTL5 (1:1000, Proteintech, 16791‐1‐AP), METTL16 (1:1000, Proteintech, 19924‐1‐AP), WTAP (1:1000, Proteintech, 10200‐1‐AP), RBM15 (1:1000, Proteintech, 10587‐1‐AP), VIRMA (1:1000, Proteintech, 25712‐1‐AP), IGF2BP2 (1:1000, Proteintech, 11601‐1‐AP), IGF2BP3 (1:1000, Proteintech, 14642‐1‐AP), YTHDC1 (1:1000, Proteintech, 14392‐1‐AP), YTHDF1 (1:1000, Proteintech, 26787‐1‐AP), YTHDF2 (1:1000, Proteintech, 24744‐1‐AP), YTHDF3 (1:1000, Proteintech, 25537‐1‐AP).Then further incubated with secondary antibodies for 1 h at room temperature, then ECL Western blotting detection regents (Biosharp, China) were used to detect proteins in imagining system (SageCreation, China).

### 2.4 RNA isolation, reverse transcription, and quantitative real‐time PCR

Total RNA was isolated from tissues and cells using TRIzol reagent (TaKaRa, Japan), then reverse transcribed to cDNA by PrimeScript™ RT reagent Kit (TaKaRa, Japan). Quantitative reverse transcription‐PCR (RT‐qPCR) reactions were conducted using the Tli RNaseH Plus kit (TaKaRa, Japan) according to manufacturers' protocols. The primers of BIRC5, METTL3, METTL14, WTAP, METTL5, METTL16, VIRMA, RBM15, YTHDC1, YTHDF1, YTHDF2, YTHDF3, IGF2BP1, IGF2BP2, and IGF2BP3 were listed in Table S1.

### 2.5 Cell transfection

Short hairpin RNAs specific for BIRC5, METTL3, IGF2BP1 and negative control, and the overexpressed plasmids (BIRC5, METTL3, METTL5, METTL14, WTAP, RBM15, RBMX, VIRMA, IGF2BP1, IGF2BP2, IGF2BP3, YTHDF1, YTHDF2, YTHDF3, and empty vector plasmid) were constructed by GeneChem (Shanghai, China). SKOV3/DDP and A2780/DDP cells were seeded into 6‐well plates with 3 × 105 cells/well concentration and transfected by Lipofectamine 3000 (Invitrogen, UA) for 48 h according to the manufacturer's protocol. In addition, cells were infected by lentivirus and selected by 1 μg/mL puromycin (Solarbio, China). The transfection efficiency was identified by RT‐qPCR and Western blotting. The sequences of shRNAs are shown in Table S2.

### 2.6 RNA stability assay

To evaluate RNA stability, OC cells who knockdown and overexpress IGF2BP1 were seeded in 12‐well plates, and treated with actinomycin D (MedChemExpress, USA) in 5 μg/mL. Total RNA was isolated after culturing for indicted times (0, 1, 3, and 6 h), and quantify the relative level of BIRC5 mRNA.

### 2.7 Dual luciferase report assay

We constructed the BIRC5 wild‐type 3′‐UTR and binding motif‐mutated 3′ vector in psiCHECK‐2 plasmid (HonorGene, China). SKOV3/DDP and A2780/DDP cells were seeded in 96‐well plates separately, and co‐transfected with wild‐type and mutated plasmids when the density reached to 70%. After incubating 48 h, we collect cells to test in Promega dual‐luciferase system (USA). The luciferase activity was normalized to the Renilla luciferase activity.

### 2.8 Me‐RIP

The RNA extracted in cell line using Total RNA Miniprep Kit (Monarch, USA), and fragmented into 300 bp with fragment reagents. Then added m^6^A anti‐body and IgG anti‐body, and added to fragment RNA into magnetic beads at 4°C for 1 h. We added RNA wash buffer to it and extracted RNA, and expression of related gene was detected by RT‐qPCR.

### 2.9 Annexin‐V‐FITC/propidium iodide staining

Annexin‐V‐FITC/propidium iodide (IP) staining was performed using Annexin‐V‐FITC/PI apoptosis assay kit (Gene‐Protein Link, China) according to its manufactures. Cells were resuspended in binding buffer, and adjusted concentration to 1 × 106. Then added 5 μL Annexin‐V‐FITC reagent to incubate for 10 min at room temperature, then added 10 μL PI reagent and 400 μL PBS to analyze by flow cytometry BD FACSCalibur (BD, Biosciences).

### 2.10 Tumor xenograft model

Four weeks old female BALB/c nude mice were purchased from SJA Laboratory Animal Co, Ltd (Changsha, China), and acclimated in a specific pathogen free environment for 1 week. SKOV3/DDP cells treated with shBIRC5, or shBIRC5 combined with pcDNA‐IGF2BP1 plasmid (IGF2BP1) or corresponding negative control were suspended in 100 μL McCoy's 5A medium and implanted subcutaneously into the left frank of mice in 1 × 10^7^ cells/mice. After 7 days incubation, three groups of mice received 10 mg/kg cisplatin respectively, sacrificing and tumor collecting after 30 days. The tumor volume measured in *V* = (L × W^2^)/2. Care and handling of the mice were approved by the Institutional Animal Care and Use Committee of Hainan Medical University (#HYLL‐2022‐218, Haikou, China).

### 2.11 Statistical analysis

All experiments were three independent repetitions, presented as mean values ±standard deviation (SD) and statistically analyzed by GraphPad Prism 9.0. Unpaired student's *t*‐test was used to evaluate the difference between experimental groups and control groups. A one‐way ANOVA test was used for comparison between three groups. Statistical significance is considered as *p < 0.05, **p < 0.01, ***p < 0.001.

## 3 RESULTS

### 3.1 Establishment of the cisplatin‐resistant cells and identification of their gene expression profile in OC

**FIGURE 1:**
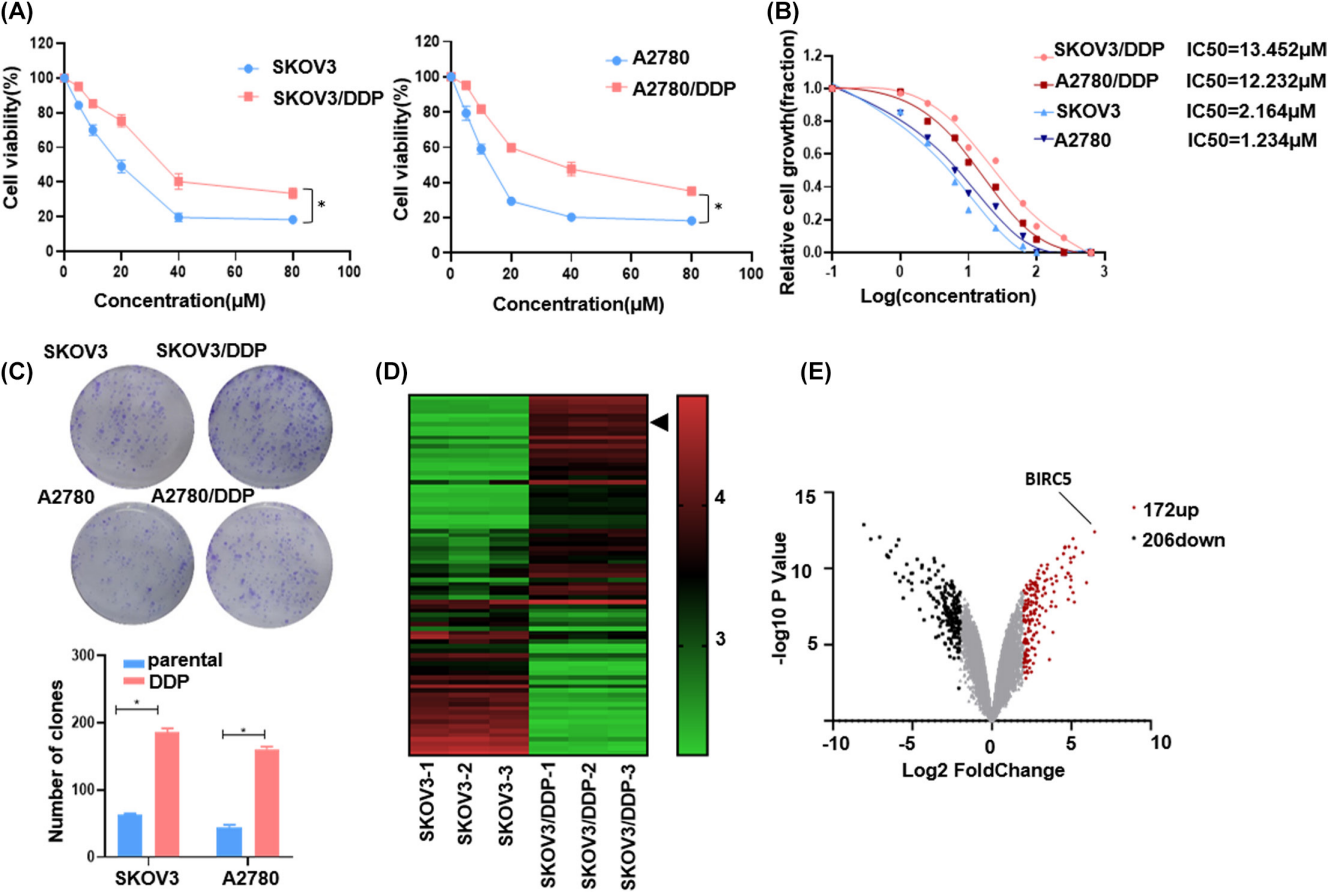
Establishment of the cisplatin‐resistant cells and identification of their gene expression profile in OC. (A) SKOV3/DDP, A2780/DDP, SKOV3 and A2780 treated with different doses of cisplatin for 24 h, followed by CCK8 assay. Dose‐response curve of cisplatin treatment in multiple OC line. (B) IC_50_ were measured after treating increasing dose of cisplatin for 24 h (log10 scaled). (C) Colony formation treated with cisplatin 20 μM for 2 weeks. (D,E) Heatmap and volcano plot represent the differentially expressed mRNA in cisplatin resistant SKOV3/DDP and counterpart SKOV3 cells. The red and green represent upregulated and down‐regulated mRNA respectively. The arrow indicates BIRC5. The mean ± SD of triplicate experiments was plotted (*n* = 3), *p < 0.05.

To unravel the potential mechanisms driving cisplatin resistance in the malignant progression of OC, we developed stable cisplatin‐resistant cell lines: SKOV3/DDP and A2780/DDP. These were derived from their respective parental cell lines by continuously exposing the cells to escalating concentrations of cisplatin. The resistant phenotypes were subsequently confirmed using the CCK8 assay. Upon cisplatin treatment, the viability of the SKOV3/DDP and A2780/DDP cells was notably higher compared to the parental SKOV3 and A2780 cells (Figure 1A). The IC_50_ values for cisplatin in the SKOV3 and A2780 cells stood at 2.164 μM and 1.234 μM, respectively. In contrast, these values surged to 13.452 μM and 12.242 μM, respectively, in the cisplatin‐resistant OC cells (Figure 1B). Concurrently, the colony formation capabilities of SKOV3/DDP and A2780/DDP surpassed those of the parental cells (Figure 1C).

To discern gene expression differences between the SKOV3/DDP and native SKOV3 cells, we executed an RNA‐sequence on total RNA. The resulting data showed fluctuations in mRNA transcript levels. Specifically, 172 RNAs were upregulated, while 206 were downregulated in the cisplatin‐resistant cells relative to the parental cells (with a fold change >2 and a *p*‐value < 0.05). Remarkably, the expression of BIRC5 exhibited the most significant increase (Figure 1D,E). Existing literature reports an overexpression of BIRC5 in numerous malignancies. This overexpression has implications for modulating carcinogenesis and chemotherapy resistance. However, the intricate molecular mechanisms underpinning these effects remain elusive.

### 3.2 BIRC5 is upregulated in OC tissues and promotes chemoresistance of OC cells in vitro

**FIGURE 2:**
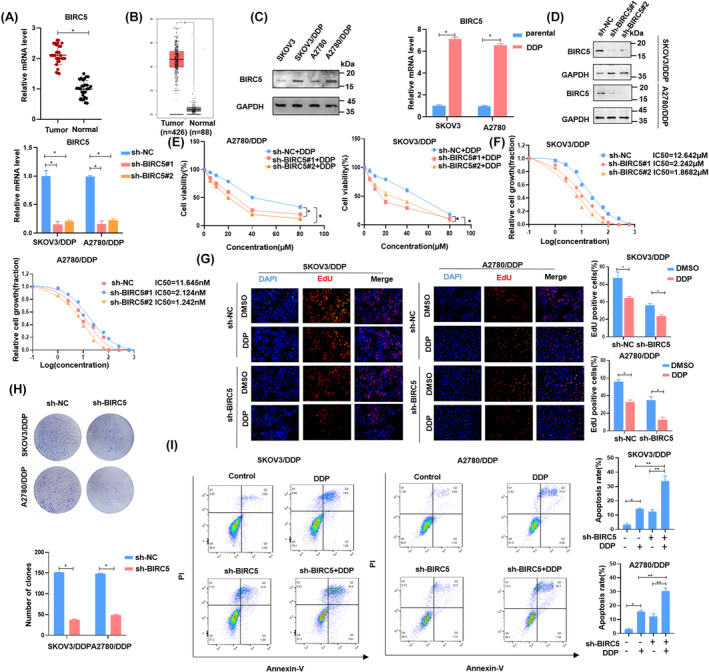
Down‐regulating BIRC5 reverses chemotherapy resistance of OC cells. (A) Relative expression of BIRC5 in OC tissues (*n* = 30) and normal ovarian tissues (*n* = 30) was detected by RT‐qPCR (*p* < 0.05). (B) The abundance of BIRC5 transcripts from the TCGA database and GTEX database. (C) Expression of BIRC5 in cisplatin resistance cell and parental cells was detected by RT‐qPCR and Western blotting. (D) SKOV3/DDP and A2780/DDP were transfected with control shRNA and BIRC5 shRNA, and the effect of knockdown BIRC5 measured by Western blotting and RT‐qPCR. (E,F) Then the cell viability monitored by CCK8, and IC_50_ after treating increasing dose of cisplatin for 24 h. (G,H) EdU and colony formation assay were used to assess cell survival of knocked‐down BIRC5 cisplatin resistance cells after 20 μM cisplatin treatment. (I) Cell apoptosis was measured by flow cytometry in SKOV3/DDP and A2780/DDP with indicated treatment, and right histograms represent apoptosis rate (early + late). The mean ± SD of triplicate experiments was plotted (*n* = 3), *p < 0.05.

To understand the role of BIRC5 in OC, we employed RT‐qPCR to measure the transcript levels of BIRC5 across 30 primary OC tissues and 30 normal ovarian tissues. As depicted in Figure 2A, the expression of BIRC5 at the mRNA level was markedly altered in primary OC tissues relative to normal ovarian tissues. This observation was congruent with the RNA‐seq datasets from TCGA and GTEx (Figure 2B).

To investigate the potential role of BIRC5 in cisplatin resistance in vitro, we assessed its expression levels in OC cells. Results indicated an upregulation of BIRC5 in SKOV3/DDP and A2780/DDP compared to the control groups (Figure 2C). Subsequently, we employed two specific shRNAs to suppress the expression of BIRC5. The effectiveness of this depletion was validated using both RT‐qPCR and Western blotting analysis (Figure 2D). Functional assays, including CCK8 and EdU, revealed that BIRC5 down‐regulation significantly amplified the sensitivity of SKOV3/DDP and A2780/DDP cells to cisplatin (Figure 2E–G). Furthermore, the IC_50_ value was diminished post‐BIRC5 silencing (Figure 2F), and the colony formation capacity was also hindered (Figure 2H).

Given that BIRC5 is an IAP, we posited that BIRC5 might attenuate cisplatin sensitivity by modulating tumor cell apoptosis. Following BIRC5 suppression, the DDP‐resistant OC cells were subjected to cisplatin treatment at an optimal dose for 24 h, after which they underwent Annexin V‐FITC/PI staining. Compared to control groups, the apoptotic rates in the BIRC5‐deficient group showed a significant increase (Figure 2I). Taken together, these findings suggested that BIRC5 played a role in modulating the sensitivity of OC cells to cisplatin.

**FIGURE 3:**
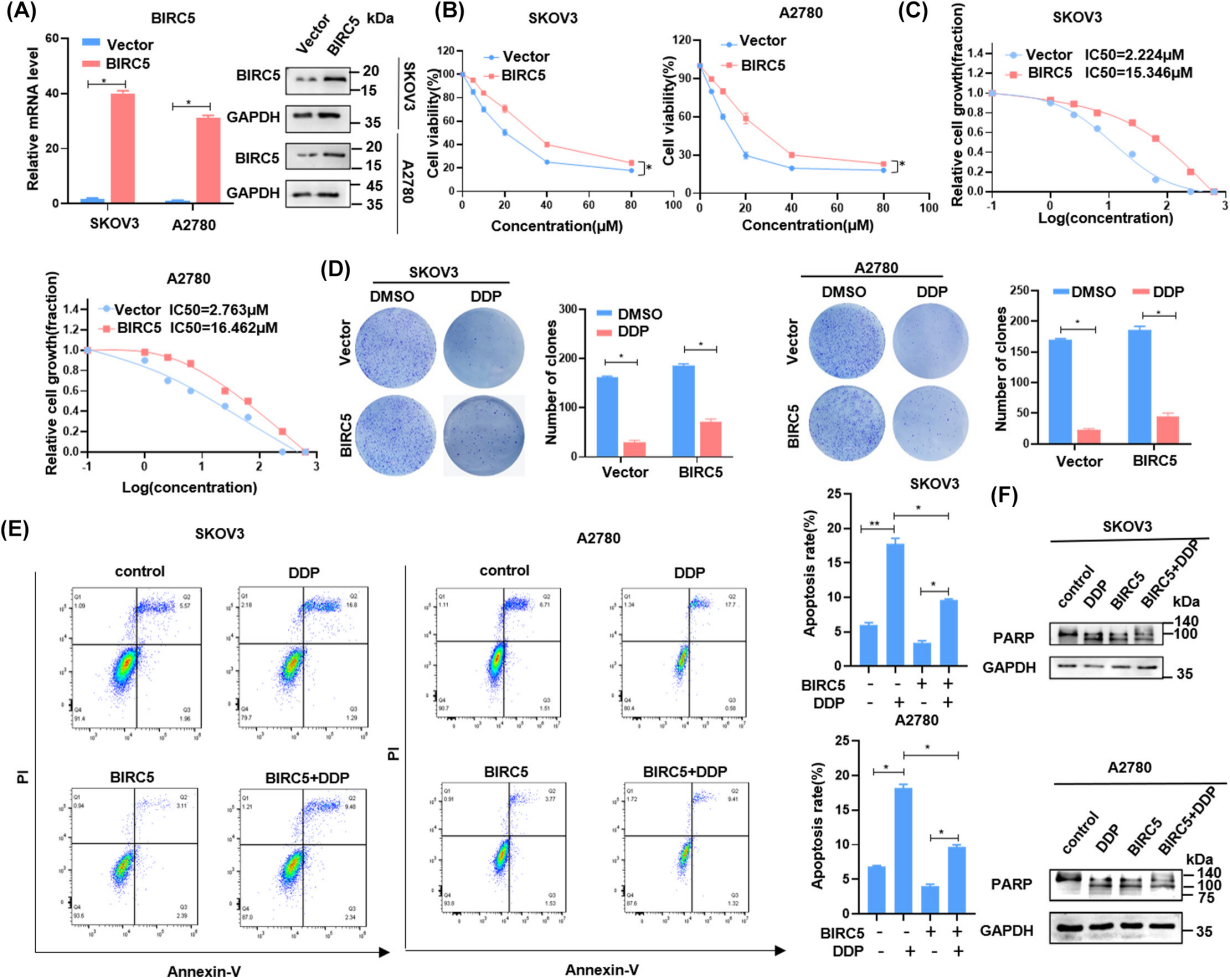
Upregulation of BIRC5 promotes cisplatin resistance of OC cells. (A) SKOV3 and A2780 cells transfected with overexpressed‐BIRC5 vector, and the effect were tested by RT‐qPCR and Western blotting. (B–D) CCK8 and colony formation assay revealed the cell viability of overexpression BIRC5 cells treated with 20 μM cisplatin, and the sensitivity to cisplatin of overexpressed BIRC5 cells measured by IC_50_. (E,F) Flow cytometry assays were performed to observe the change of percentage of apoptosis cells (early + late) after upregulating BIRC5, and the expression level of apoptosis biomarker PARP was detected by Western blotting. The mean ± SD of triplicate experiments was plotted (*n* = 3), *p < 0.05.

Simultaneously, we generated and transfected a BIRC5 plasmid into SKOV3 and A2780 cells. The effectiveness of this transfection was validated using both RT‐qPCR and Western blotting analysis (Figure 3A). Enhanced BIRC5 expression augmented cell viability and proliferation while diminishing cisplatin sensitivity, as evidenced by CCK8, IC_50_, and colony formation experiments (Figure 3B–D). Moreover, elevating BIRC5 levels resulted in reduced apoptotic rates, as assessed by flow cytometry analyses alongside cleaved‐PARP (Figure 3E,F). In summary, these observations reinforced the notion that BIRC5 was pivotal for conferring cisplatin resistance in OC cells.

### 3.3 BIRC5 is regulated by METTL3‐mediated m^6^A modification

**FIGURE 4:**
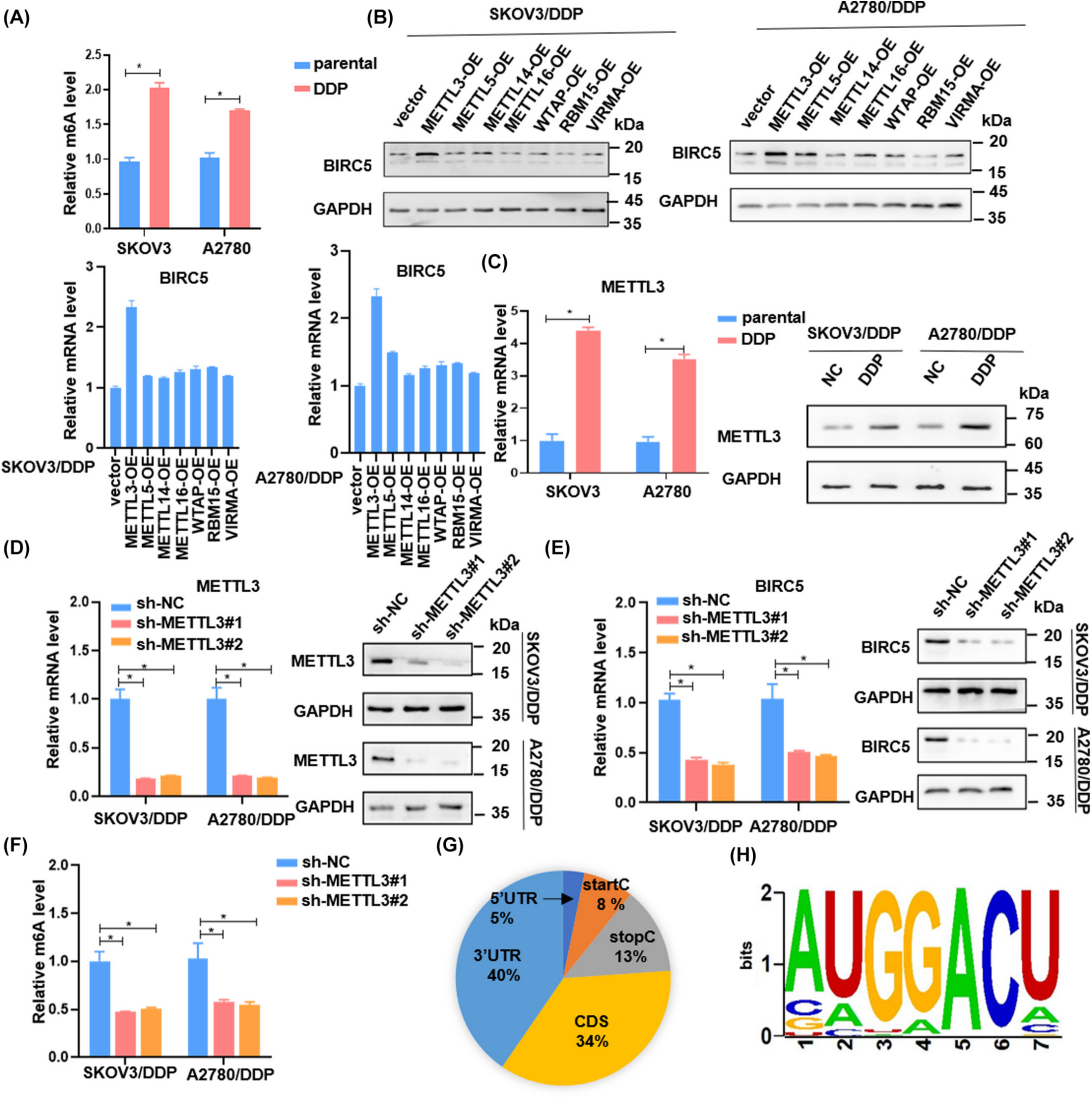
BIRC5 is regulated by METTL3‐mediated m^6^A modification. (A) RNA extracted in SKOV3, SKOV3/DDP, A2780 and A2780/DDP, and m6A status was examined by 450 nm absorbance according to m^6^A methylation assay kit manuscript (Abcam, USA). (B) Overexpression of predicted writers and measure the expression of BIRC5 in mRNA and protein level. (C) The mRNA and protein expression level of METTL3 in OC cells. (D–F) The effect of silenced METTL3, with the decreased BIRC5 expression level and m^6^A level. (G) MeRIP‐seq confirmed the m^6^A peak location. (H)Predicted binding motif of METTL3 on 3′UTR of BIRC5 mRNA from RMBase website. The mean ± SD of triplicate experiments was plotted (*n* = 3), *p < 0.05.

Prior research has indicated that m^6^A modification plays an important role in drug resistance across various tumors. To understand the molecular mechanism through which BIRC5 influences chemotherapy resistance in OC, we compared the m^6^A levels between parental SKOV3, A2780 cells, and their cisplatin‐resistant counterparts: SKOV3/DDP and A2780/DDP. Colorimetric analysis for m^6^A RNA methylation quantification revealed that m^6^A levels in mRNAs from SKOV3/DDP and A2780/DDP cells were significantly elevated compared to those in SKOV3 and A2780 cells (Figure 4A). Based on these findings, we hypothesized that m^6^A modification might play a part in regulating BIRC5 in cisplatin‐resistant cells.

To test this hypothesis, we assessed the correlation between m^6^A methyltransferases and BIRC5. Data from RT‐qPCR and Western blotting analysis showed that, upon overexpression of the methyltransferases, only METTL3 consistently influenced the expression of BIRC5 in DDP‐resistant OC cells (Figure 4B, Figures S1 and S2). Thus, we focused on the well‐established m^6^A reader METTL3, which was also markedly upregulated in SKOV3/DDP and A2780/DDP cells in comparison to their respective control cells (Figure 4C).

To investigate the role of METTL3 in modulating BIRC5 in OC chemoresistance and malignant progression, we suppressed METTL3 expression using shRNA (shMETTL3) in SKOV3/DDP and A2780/DDP cells (Figure 4D). Both RT‐qPCR and Western blotting analysis revealed that METTL3 positively influenced the expression of BIRC5 in DDP‐resistant OC cells (Figure 4E). Additionally, the depletion of METTL3 notably reduced m^6^A modification levels (Figure 4F).

To further confirm the regulatory role of METTL3 in OC chemoresistance, we used the SRAMP tool (http://www.cuilab.cn/sramp/) to predict m^6^A modification sites on BIRC5. The tool highlighted that BIRC5 possessed multiple m^6^A sites (Figure S3). MeRIP‐seq analysis of m^6^A modifications in control and METTL3‐depleted SKOV3/DDP cells showed that m^6^A peaks were predominantly situated near the 3′‐UTR (Figure 4G). Furthermore, the common motif of these peaks were identified as DRACH by the RMBase website (Figure 4H). This finding suggested that METTL3 might modulate target gene expression via methylation modification in OC. This mechanism could be a primary factor for the elevated BIRC5 expression in chemotherapy‐resistant OC cells relative to control cells.

### 3.4 IGF2BP1 promotes OC patients' chemotherapy resistance

**FIGURE 5:**
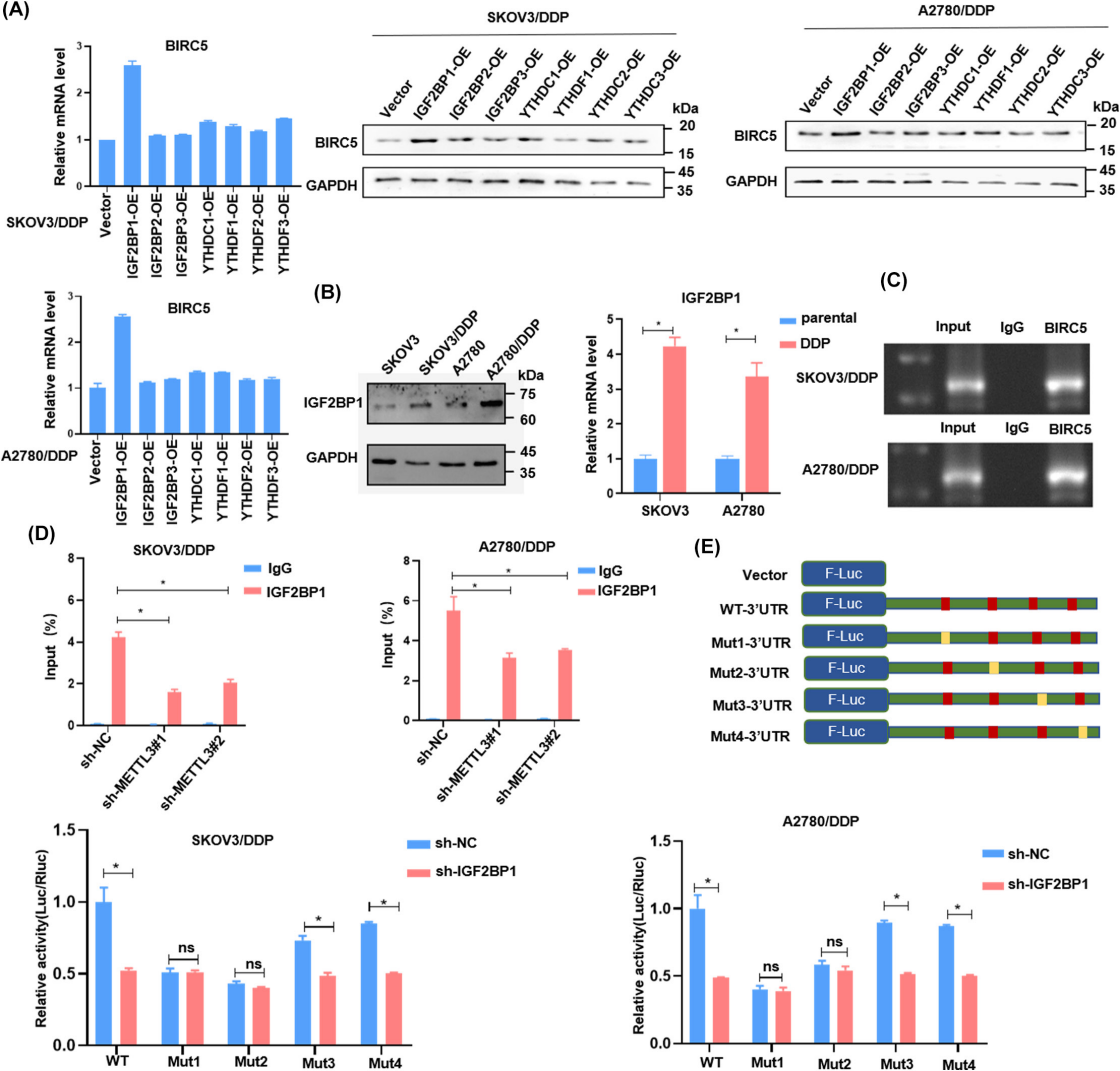
IGF2BP1 promotes OC patients' chemotherapy resistance. (A) BIRC5 expression level in mRNA and protein after overexpressed “readers”. (B) The expression of IGF2BP1 in cisplatin‐resistant cells and counterpart cells. (C–D) RIP assay was used to detect the ability of IGF2BP1 binding to BIRC5. (E,F) Point mutation in m6A modification motif with lower luciferase reporter activities, and mutated BIRC5 with silenced IGF2BP1 was represented to explore the m6A roles in BIRC5 expression. The mean ± SD of triplicate experiments was plotted (*n* = 3), *p < 0.05, ns was not statistically significant.

According to recent research, the IGF2BP protein family has the ability to bind m^6^A‐modified mRNA, which plays a pivotal role in RNA stability. We utilized m^6^A2Target (http://m6a2target.canceromics.org/) to predict potential targets, as illustrated in Figure S4. To validate the regulatory relationship with BIRC5, we overexpressed reader proteins in both SKOV3/DDP and A2780/DDP cells. Our findings demonstrated that upregulating IGF2BP1 significantly augmented the expression of BIRC5 at both the mRNA and protein levels (Figure 5A; Figure S5). Moreover, there was a notable upregulation of IGF2BP1 in SKOV3/DDP and A2780/DDP cells compared to control cells (Figure 5B).

We conducted an RNA immunoprecipitation (RIP) assay in DDP‐resistant OC cells to confirm the interaction between IGF2BP1 and BIRC5 mRNA. Our results indicated that BIRC5 mRNA was distinctly enriched when using the anti‐IGF2BP1 antibody. This enrichment was markedly reduced upon METTL3 depletion (Figure 5C,D).

To decipher the role of IGF2BP1 in cisplatin resistance, we carried out luciferase assays. The luciferase activity of wild‐type (WT) or mutant (Mut) 3′UTR sequences was analyzed in SKOV3/DDP and A2780/DDP cells. Compared to cells with the WT construct, those transfected with the Mut construct exhibited a pronounced decrease in luciferase reporter activity. This finding suggested that IGF2BP1 played a crucial role in stabilizing mRNAs with WT sequences. Furthermore, when comparing the Mut to the WT, the luciferase activity in the control group showed a decrease. However, upon silencing IGF2BP1, there was no significant change in luciferase activity, indicating that the primary binding sites were site 1 and site 2 (Figure 5E; Figure S6).

### 3.5 METTL3/IGF2BP1/BIRC5 axis is essential for cisplatin resistance of OC

**FIGURE 6:**
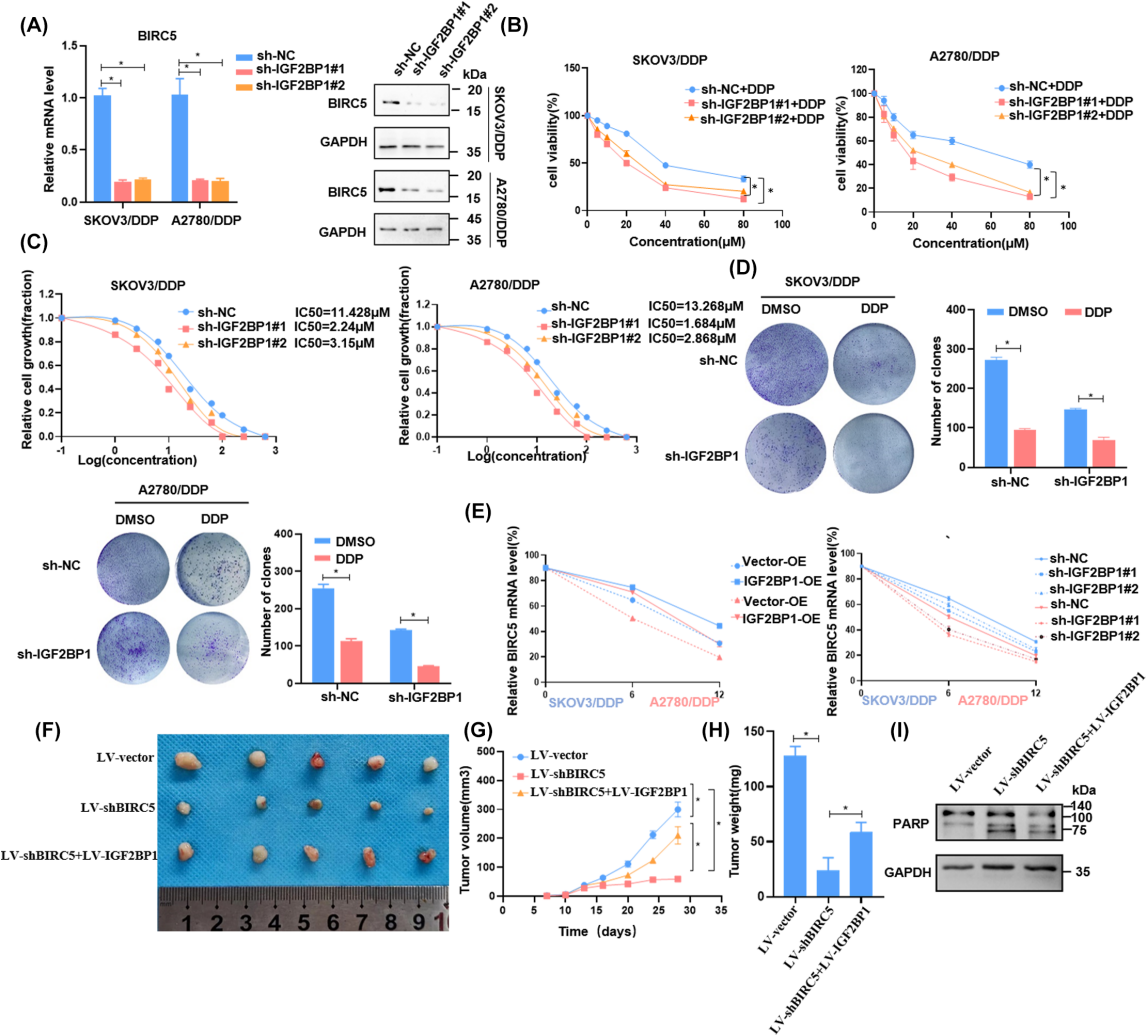
METTL3/IGF2BP1/BIRC5 axis is essential for cisplatin resistance of OC. (A) The expression level of BIRC5 in silenced IGF2BP1 OC cells. (B–D) Cell viability detected by CCK8, the sensitivity of cisplatin detected by IC_50_, and the clonogenic ability in silenced IGF2BP1 OC cells (left panel), quantification of the colony formation assay results (right panel). (E) The mRNA expression of BIRC5 was measured by RT‐qPCR in SKOV3/DDP and A2780/DDP, who overexpressed or silenced IGF2BP1 upon actinomycin D (ActD) treatment for 8 h. (F–I) Nude mice bearing SKOV3/DDP cells treated with shBIRC5, or shBIRC5 combined with pcDNA‐IGF2BP1 plasmid (IGF2BP1) or corresponding negative control, and after 7 days the mice treated with cisplatin (10 mg/kg). Tumor volume were measured and record 4 days after injection. Tumor were removed after 28 days, and their weights had significantly differences. The protein expression of PARP was measured by Western blotting. The mean ± SD of triplicate experiments were plotted (*n* = 3), *p < 0.05,**p < 0.01.

To further investigate the role of IGF2BP1 in OC chemotherapy resistance, we transfected SKOV3/DDP and A2780/DDP cells with sh‐IGF2BP1. The silencing of IGF2BP1 led to a marked reduction in both mRNA and protein expression levels of BIRC5 (Figure 6A). Through CCK8 and colony formation assays, we found that the downregulation of IGF2BP1 significantly increased the sensitivity of the cells to cisplatin and reduced the IC_50_ value in both SKOV3/DDP and A2780/DDP cells (Figure 6B–D). To corroborate these findings, we conducted an RNA decay assay. SKOV3/DDP and A2780/DDP cells were treated with actinomycin D, an RNA transcription inhibitor, at different intervals. Results indicated that BIRC5 mRNA remained highly stable upon IGF2BP1 overexpression. Conversely, a decrease was observed in the cells where IGF2BP1 was depleted (Figure 6E).

To further explore the roles of the METTL3/IGF2BP1/BIRC5 axis in OC in vivo, we subcutaneously implanted BALB/c nude mice with SKOV3/DDP cells. These cells were stably transduced with either a lentiviral vector containing a negative control sequence (LV‐vector), a shBIRC5 sequence (LV‐shBIRC5), or a combination of lentivirus containing IGF2BP1 and shBIRC5 (LV‐shBIRC5 + LV‐IGF2BP1). After 7 days incubation, three groups of mice received 10 mg/kg cisplatin respectively, and we measured the xenograft volumes in mice every 4 days. Our observations indicated that the depletion of BIRC5 substantially reduced the tumor volume. However, this effect could be offset by the co‐treatment involving IGF2BP1 overexpression (Figure 6F,G). After sacrificing the mice, we assessed the tumor weights, which aligned with our volume findings (Figure 6H). Furthermore, tumors treated with LV‐shBIRC5 exhibited elevated PARP protein expression—a well‐known marker for apoptosis. This effect was mitigated upon co‐treatment with LV‐IGF2BP1 overexpression (Figure 6I). Collectively, these results underscored that IGF2BP1 contributed to cisplatin resistance by modulating BIRC5 mRNA stability, emphasizing the importance of the METTL3/IGF2BP1/BIRC5 axis in OC cisplatin resistance.

## 4 DISCUSSION

We uncovered the potential role and function of BIRC5 in cisplatin resistance in OC through an m^6^A‐dependent mechanism. The resistance to chemotherapy remains a significant hurdle in OC treatment, making it crucial to comprehend its underlying mechanisms. While prior research has highlighted the upregulation of BIRC5 in various tumors,^19^, ^20^, ^21^ our study delved deeper to show that BIRC5 regulation was associated with RNA methylation involving specific methyltransferases. Depleting BIRC5 not only heightens sensitivity to cisplatin but also escalates the rate of cisplatin‐induced apoptosis.

Bioinformatics analyses indicated that BIRC5 had abundant m^6^A binding sites, pinpointing METTL3 and IGF2BP1 as potential regulators. Utilizing methods like meRIP‐seq, RIP assays, and luciferase assays, we validated that METTL3 and IGF2BP1 could indeed modulate the expression level of BIRC5 and its RNA stability in an m^6^A‐dependent manner. Moreover, in vivo experiments, wherein tumors were transplanted into BALB/c nude mice, corroborated these findings. Notably, we observed distinct differences in tumor weight and volume among the control group, the BIRC5 knockdown group, and the group with BIRC5 knockdown combined with IGF2BP1 overexpression.

BIRC5, also known as survivin, belongs to the IAP family. It encodes proteins that act as negative regulators, inhibiting cell death. Aberrant expression of BIRC5 has been documented in various malignancies. This expression has been directly linked to aspects of cancer progression such as tumor growth, invasion, metastasis, poor prognosis, immune infiltration, and resistance to chemotherapy.^22^, ^23^ BIRC5 is central to several signaling pathways. Consequently, understanding the upstream molecules that regulate its expression and function has become a focal point in cancer therapy research. These regulatory molecules encompass a wide range, including miRNAs, transcription factors, binding proteins, and protein regulators. For instance, there is emerging evidence suggesting that USP1 enhances BIRC5 stabilization through ubiquitin, which significantly prevents apoptosis induced by ML323 and TRAIL.^24^ Suppression of both Mcl‐1 and BIRC5 has been shown to efficiently curtail cell growth and increase drug sensitivity.^25^ In the context of OC, studies have indicated that miR‐203 targets BIRC5, suppressing OC metastasis.^26^ Furthermore, low‐dose radiation has been found to reverse cisplatin resistance by down‐regulating BIRC5.^27^ Although BIRC5 plays an instrumental role in chemoresistance, its precise underlying mechanism remains elusive.

Cisplatin, known for its potent antitumor efficacy, is a primary chemotherapeutic drug for OC. However, the onset of chemoresistance poses a significant challenge for OC treatment. Cisplatin binds to the DNA of cancer cells, facilitating both intra‐chain and inter‐chain cross‐linking. When damage from this process is irreparable, it induces apoptosis. Chemoresistance mechanisms vary based on their sites and timeframes and can be categorized as pre‐target, target, post‐target, and off‐target drug resistance.

Among these, epigenetic modifications form part of the off‐target drug resistance mechanisms. These modifications span DNA, RNA, and protein adjustments. Notably, RNA methylation has emerged as a focal point in recent research. m^6^A, known for its role in post‐transcriptional regulation, has diverse functions in cancer progression.^28^, ^29^ Many studies have extensively explored METTL3 across various cancer types. METTL3, an m^6^A “writer”, can independently or collaboratively regulate aspects of cancer, such as growth, invasion, metastasis, and drug resistance.^30^, ^31^, ^32^, ^33^ For instance, it's been reported that METTL3 partners with YTHDF1 to boost hepatocellular carcinoma progression,^34^ triggers the PI3K/AKT signaling pathway to enhance prostate cancer growth,^35^ and plays roles in chemoresistance across various cancers like oral cancer,^36^ acute myeloid leukemia,^37^ and more.

In this study, using MeRIP‐seq and RIP assays, we determined that METTL3 activated BIRC5, leading to increased expression levels and decreased cisplatin sensitivity. The further analysis predicted potential RBPs binding to BIRC5. Subsequent tests, including RIP and dual luciferase reporter assays, identified IGF2BP1 as a stabilizer of BIRC5 mRNA. Recognized as a “reader” of RNA‐binding proteins, our research substantiated IGF2BP1's role in stabilizing BIRC5 mRNA. It collaborated with METTL3 to modulate drug resistance, as evidenced by our cellular biology experiments.

In conclusion, we found a significant elevation of BIRC5 in cisplatin‐resistant OC cell lines. Our research further illuminated that METTL3 and IGF2BP1 contributed to chemoresistance: METTL3 by enhancing BIRC5 expression and IGF2BP1 by stabilizing BIRC5 mRNA through RNA methylation.

## AUTHOR CONTRIBUTIONS

**Yadan Fan:** Formal analysis (equal); methodology (equal); writing – original draft (equal). **Yinglian Pan:** Data curation (equal); supervision (equal). **Liping Jia:** Supervision (equal); visualization (equal). **Shuzhen Gu:** Validation (equal). **Binxin Liu:** Data curation (equal); resources (equal). **Ziman Mei:** Visualization (equal). **Chunyan Lv:** Software (equal). **Haohao Huang:** Supervision (equal). **Genhai Zhu:** Investigation (equal); project administration (equal). **Qingchun Deng:** Funding acquisition (equal); resources (equal).

## FUNDING INFORMATION

This study was supported by the Hainan Provincial Key Research and Development. Program Project Fund (No. ZDYF2022SHFZ068), the High‐level Talents Program of. Hainan Province Natural Science Foundation (No. 821RC712), the Hainan Health. Commission Project (No. 20A200247), the National Natural Science Foundation of China (No. 82203879), the China Postdoctoral Science Foundation (No. 2021MD703960), Nature Science Foundation of Hubei Province (No. 2022CFB883), and General Hospital of Central Theater Command of Chinese People's Liberation Army Foundation (No. ZZYCZ202107).

## CONFLICT OF INTEREST STATEMENT

The authors declare that they have no competing interests.

## ETHICS STATEMENT

Written informed consent was obtained from all the participants prior to the enrollment of this study. And all experiments were conducted in accordance with the ethical standards of the ethics committee of The Second Affiliated Hospital, Hainan Medical University.

## Supporting information


Figure S1.

Figure S2.

Figure S3.

Figure S4.

Figure S5.

Figure S6.

Table S1.

Table S2.

## Data Availability

All data generated or analyzed during the present study are included in this published article or are available from the corresponding author on reasonable request.
