# Supplementary figures and images for: BIRC5 facilitates cisplatin‐chemoresistance in a m6A‐dependent manner in ovarian cancer
Source: Cancer Med. 2023 Dec 19;13(1):e6811. doi: 10.1002/cam4.6811 (PMC10807614; doi:10.1002/cam4.6811)

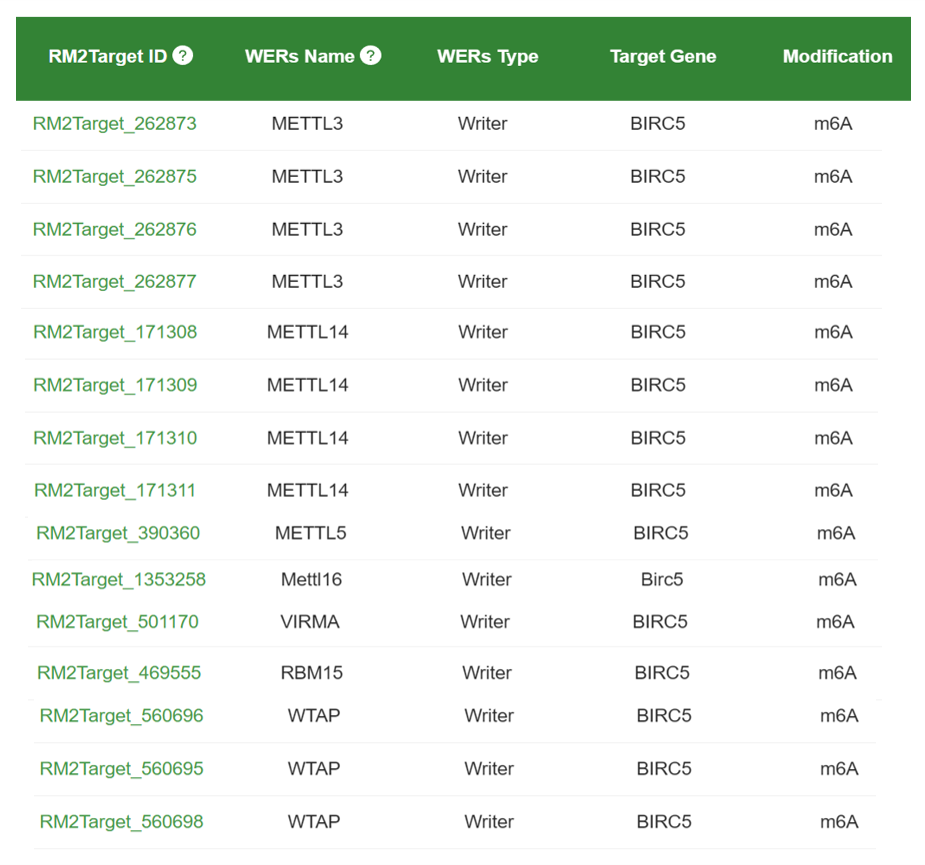

Supplement: Supplementary file 1 — Figure S1. [file CAM4-13-e6811-s006.tif]

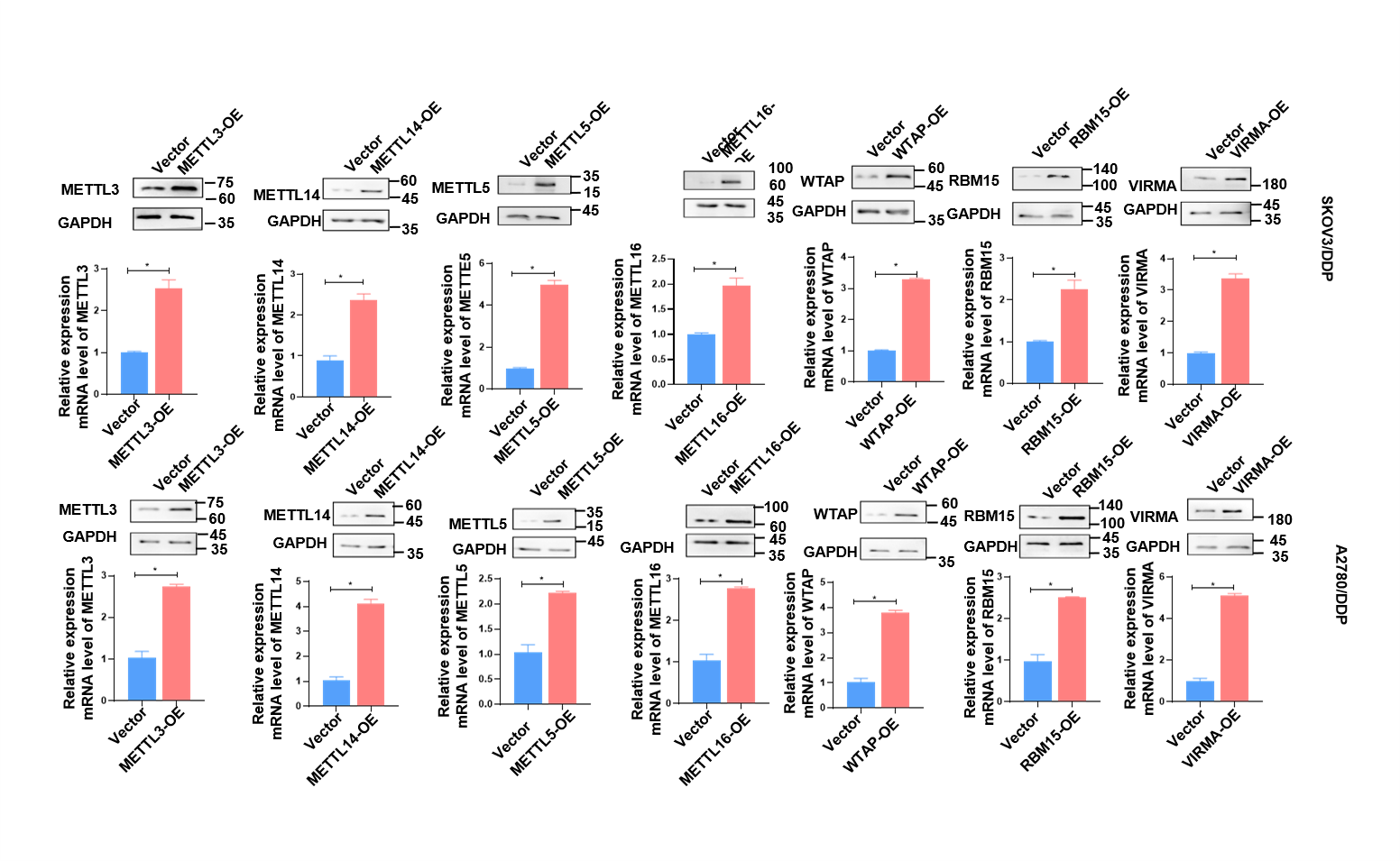

Supplement: Supplementary file 2 — Figure S2. [file CAM4-13-e6811-s005.tif]

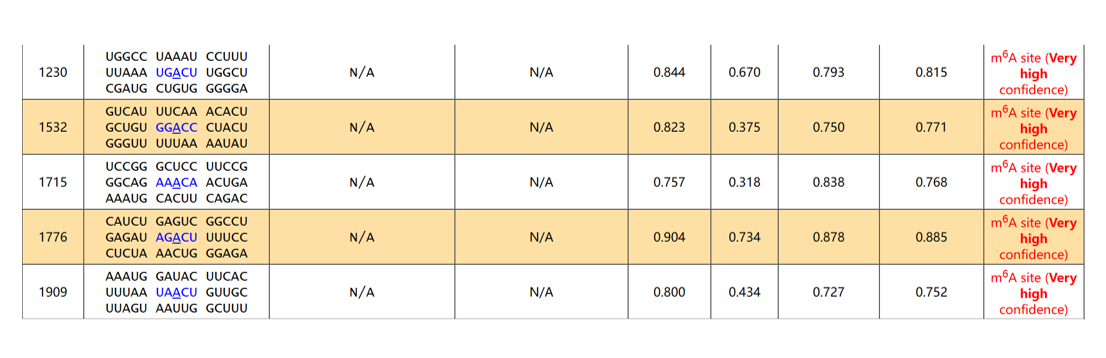

Supplement: Supplementary file 3 — Figure S3. [file CAM4-13-e6811-s003.tif]

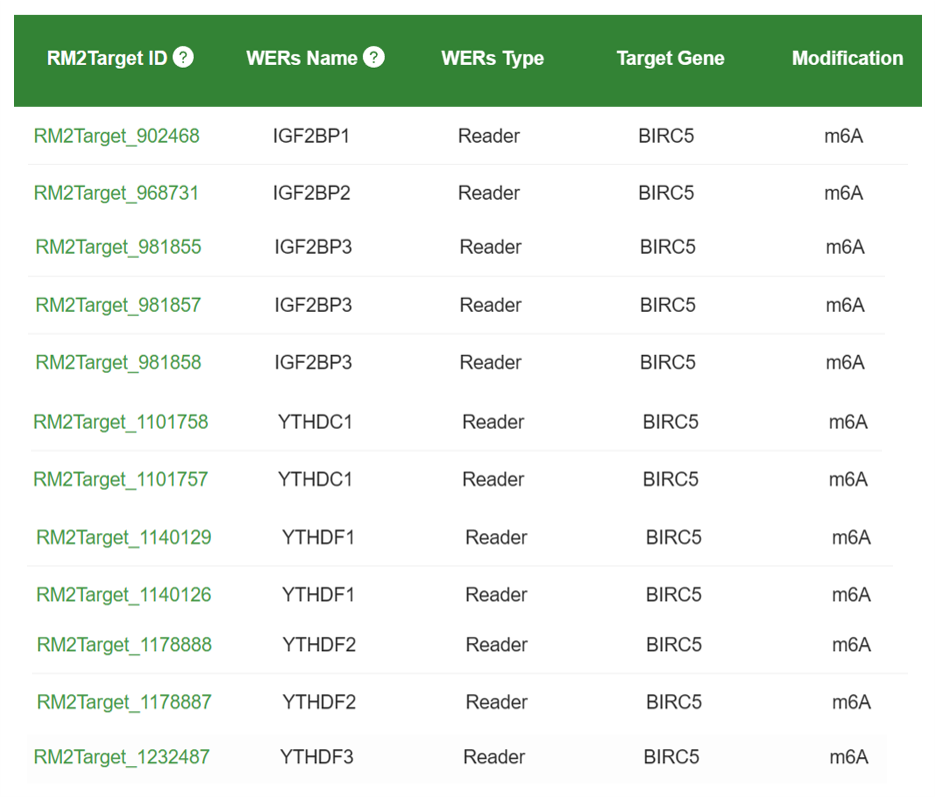

Supplement: Supplementary file 4 — Figure S4. [file CAM4-13-e6811-s008.tif]

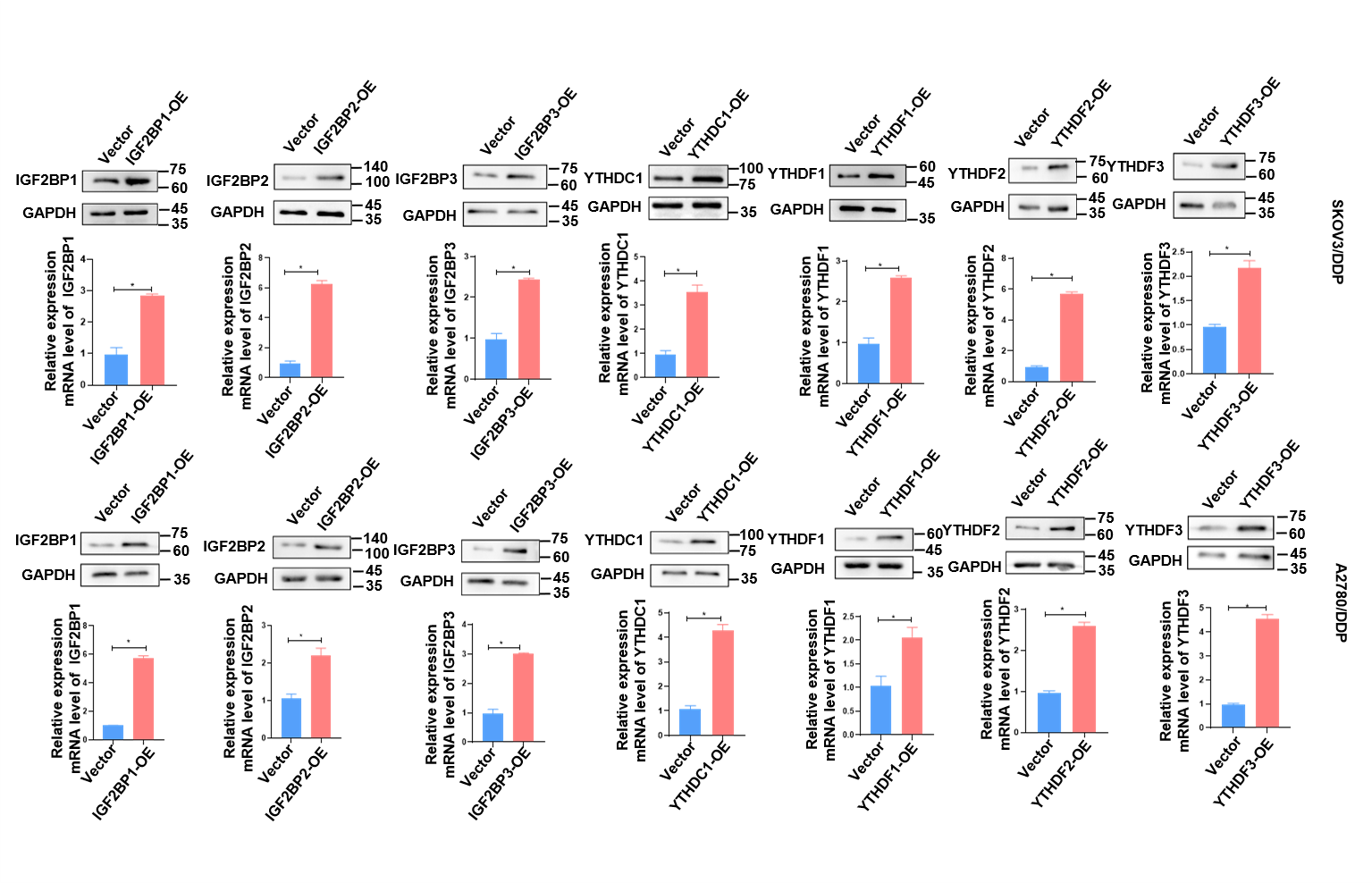

Supplement: Supplementary file 5 — Figure S5. [file CAM4-13-e6811-s007.tif]

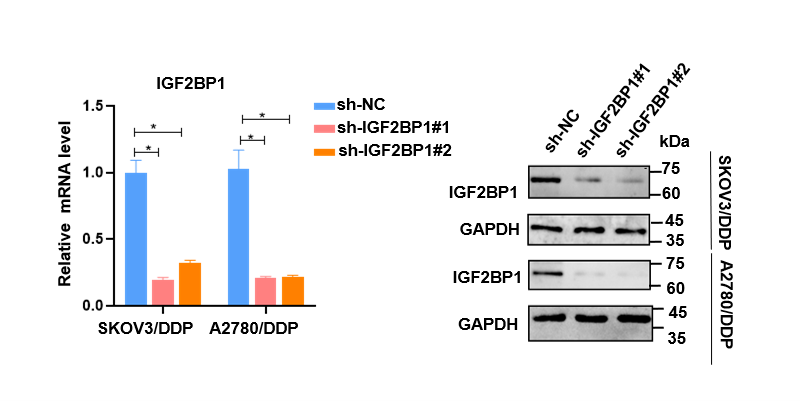

Supplement: Supplementary file 6 — Figure S6. [file CAM4-13-e6811-s004.tif]

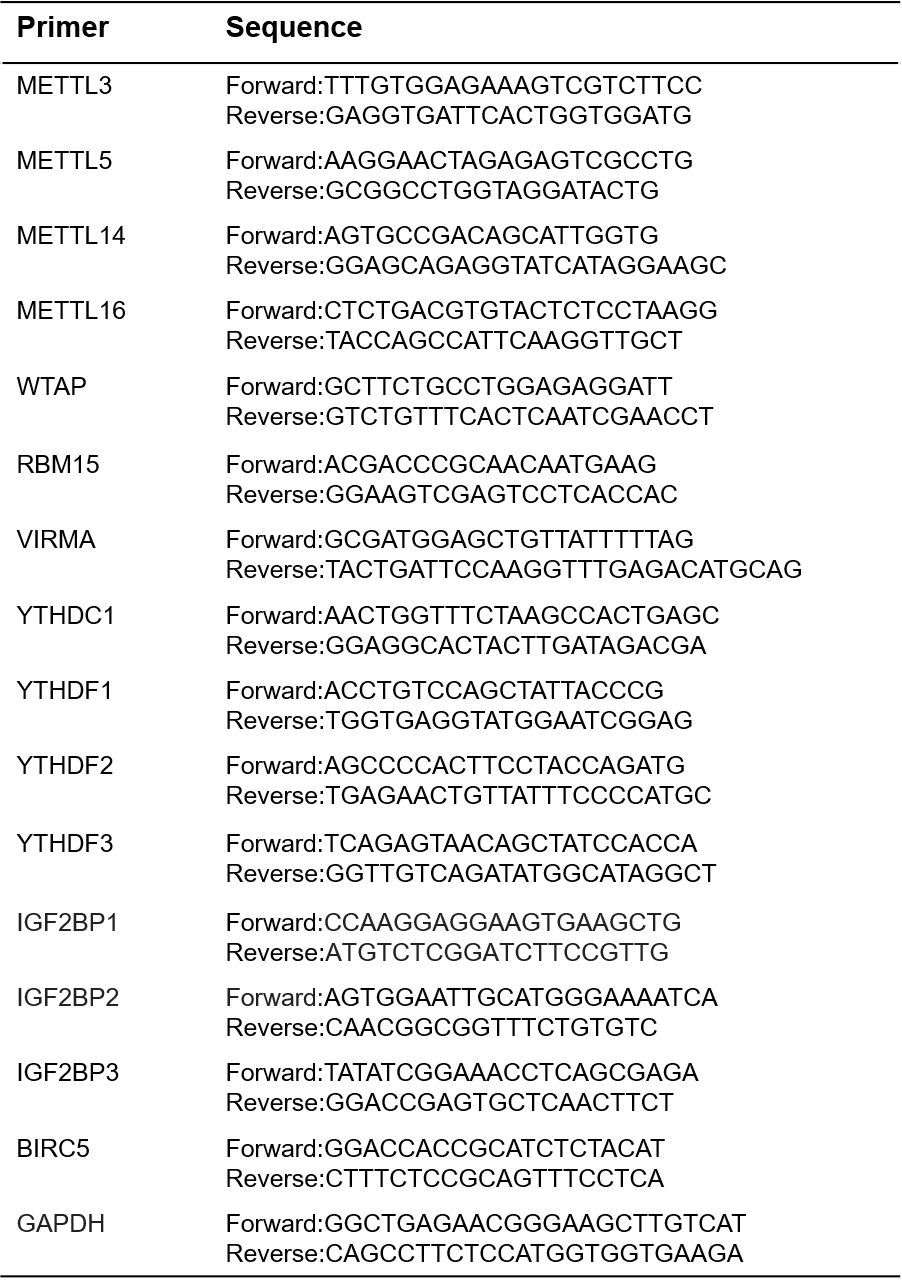

Supplement: Supplementary file 7 — Table S1. [file CAM4-13-e6811-s001.tif]

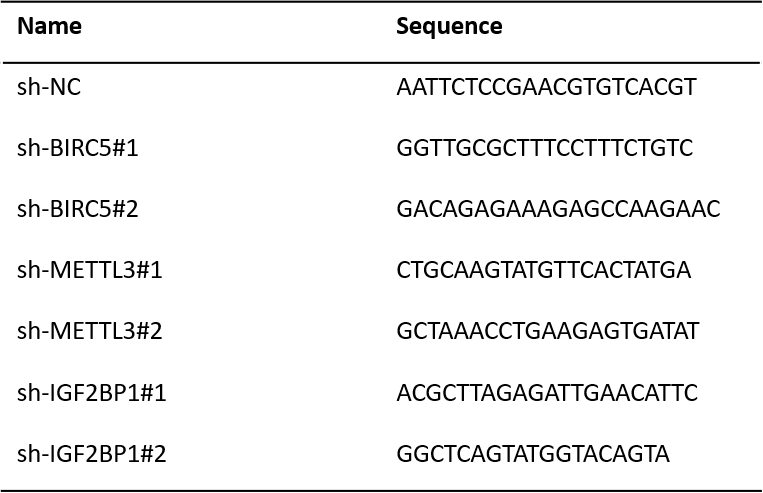

Supplement: Supplementary file 8 — Table S2. [file CAM4-13-e6811-s002.tif]
